# Effect of spray COAG mode on hemostasis in colorectal endoscopic submucosal dissection using inverse probability of treatment weight analysis

**DOI:** 10.1002/deo2.70008

**Published:** 2024-09-16

**Authors:** Jun Kanazawa, Hisatomo Ikehara, Toshiki Horii, Gen Kitahara, Tomohiro Betto, Kaoru Yokoyama, Kiyonori Kobayashi, Chika Kusano

**Affiliations:** ^1^ Department of Gastroenterology, Internal Medicine Kitasato University School of Medicine Kanagawa Japan

**Keywords:** colon cancer, ESD, hemostasis, PECS, VIO3

## Abstract

**Objective:**

Swift and forced COAG with an electrosurgical knife are commonly used for intraoperative hemostasis in colorectal endoscopic submucosal dissection (ESD). If bleeding cannot be stopped using an electrosurgical knife, cauterization is attempted using hemostatic forceps. Since April 2022, our hospital has started using Spray COAG for intraoperative hemostasis for colorectal ESD. This study aimed to provide evidence of the efficacy of Spray COAG for intraoperative hemostasis.

**Methods:**

Colorectal ESD was performed for 320 lesions at our hospital. Of these, 307 were included; 145 and 162 lesions were operated before and after the introduction of Spray COAG, respectively. Spray COAG was used after the change. The primary endpoint was the change in the frequency of use of hemostatic forceps after the introduction of Spray COAG; the secondary endpoint was the change in the prevalence of postoperative complications after the introduction of Spray COAG. It should be noted that the Spray COAG mode was employed solely for hemostasis and not for dissection, while the Swift COAG mode was utilized for dissection in the After Spray COAG group. Statistical analysis was conducted using IPTW analysis.

**Results:**

The frequency of use of hemostatic forceps was significantly decreased after the introduction of Spray COAG (odds ratio = 0.12, 95% confidence interval [95%CI]: 0.06–0.23, *p* < 0.001). The prevalence of post‐ESD electrocoagulation syndrome significantly decreased (odds ratio = 0.43, 95%CI: 0.22–0.88, *p* = 0.02). No significant differences were observed between the intraoperative and postoperative perforations or rate of postoperative bleeding.

**Conclusion:**

Spray COAG reduced the frequency of hemostatic forceps use in colorectal ESD.

## INTRODUCTION

Endoscopic submucosal dissection (ESD) involves the use of a small electrosurgical knife to incise the mucosa and dissect the submucosal layer for colorectal tumors. It was developed by Ono et al. for en‐bloc resection of lesions.^1^ ESD has become popular globally primarily as an en‐bloc resection method for gastric cancer. In contrast, Uraoka et al. reported the multicentric infiltration of the submucosa by a laterally spreading tumor (LST) while exploring the application of ESD to colonic lesions.^2^ En‐bloc resection of the large intestine using ESD, mainly for LST, has become widely practiced following this report.^3^, ^4^ Furthermore, relatively large‐diameter arteries and veins run through the submucosal layer, and intraoperative bleeding control is important when performing ESD for large LSTs.^5^ Methods to stop bleeding during colorectal ESD include using an electrosurgical knife and the coagulation mode of a high‐frequency surgical device, grasping the blood vessel using hemostatic forceps, and using non‐energized methods such as clipping and hemostatic agents.

VIO3 (Erbe) is the latest high‐frequency power supply developed by Erbe.^6^ It was launched in Japan in 2017 and is widely used in the surgical and endoscopy fields.^3^ VIO3 measures tissue resistance 25 million times per second to maintain the appropriate high‐frequency output. It has four coagulation modes: Swift COAG, Forced COAG, Spray COAG, and Soft COAG. Conventionally, for intraoperative hemostasis using an electrosurgical knife in colorectal ESD, Swift COAG or Forced COAG is used as the coagulation mode. However, the current situation is that Erbe has not specified the recommended settings for VIO3 for colorectal ESD. Therefore, there is variation among physicians regarding the optimal mode setting and power adjustment of the VIO3 to control intraoperative bleeding during colorectal ESD. Hemostasis is performed using hemostatic forceps when bleeding cannot be stopped using an electrosurgical knife.

Since April 2022, our hospital has reviewed the mode settings of VIO3 and started using Spray COAG, which has higher coagulability for intraoperative bleeding than other settings.

After adopting Spray COAG, we realized that the frequency of use of hemostatic forceps for intraoperative bleeding had decreased. We hypothesized that the use of Spray COAG for intraoperative bleeding control during colorectal ESD would decrease the frequency of the use of hemostatic forceps. This study aimed to provide evidence of the efficacy of Spray COAG for intraoperative hemostasis.

## METHODS

### Ethics statement

This study was approved by the Ethics Review Board of the Kitasato University School of Medicine and Hospital (approval number: B22‐228), and the opt‐out method was used to obtain consent from the study participants.

### Study design and population

We performed colorectal ESD for LSTs at Kitasato University Hospital from April 1, 2020, to August 31, 2023, and targeted 320 lesions for which en‐bloc resection and treatment were completed. In this single‐center, retrospective study, information on the ESD instruments and procedures was collected from the endoscopic records, and the electronic medical records were accessed to record post‐ESD physical findings and blood test results.

**FIGURE 1:**
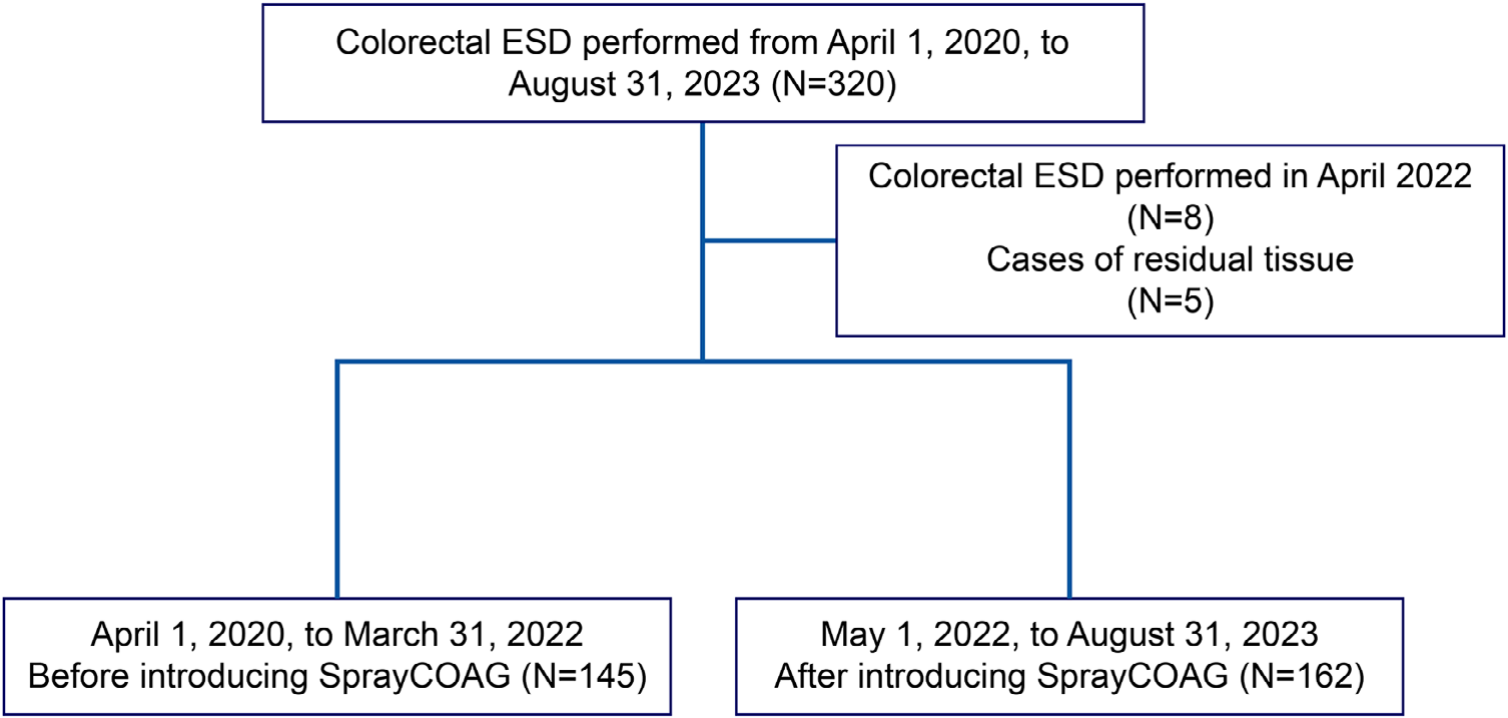
The selection of the participants. ESD, endoscopic submucosal dissection.

Figure 1 shows the selection of the participants. A total of 307 of 320 lesions that had undergone ESD between April 2020 and August 2023 were included. Eight lesions in patients who had undergone ESD between April 1 and 31, 2022, the period during which the new VIO3 setting was explored, were excluded. Five lesions with residual recurrence after endoscopic treatment were also excluded. Finally, 145 and 162 lesions were included before and after the introduction of Spray COAG, respectively.

### Definitions

LST is defined as a tumor with a diameter of 10 mm or more that is mainly characterized by lateral growth. Its macroscopic types are granular (LST granular type: LST‐G) and non‐granular (LST non‐granular type: LST‐NG). Pathologically, it is broadly divided into benign (adenoma) and malignant (adenocarcinoma), and the depth of invasion of malignant tumors is classified as pTis/pT1a and pT1b or deeper on the basis of the 9th edition of the Japanese Classification of Colorectal, Appendiceal, and Anal Carcinoma.^7^

The tumor sites were the colon (cecum to sigmoid colon) and rectum. Based on the report by Chiba et al.,^8^ depending on the number of ESDs performed by the physician, 60 or more cases were categorized as professional, and 59 or fewer cases were categorized as beginner. ESD was performed by beginners under professional supervision for all the cases.

Postoperative bleeding after ESD was defined in this study as bleeding that necessitated additional endoscopic hemostatic procedures. Cases of minor bleeding were excluded from this definition, as distinguishing them from postoperative bleeding due to the drainage of residual intraoperative blood is difficult, and such minor bleeding does not significantly affect the clinical course.

Post‐ESD electrocoagulation syndrome (PECS) was defined as abdominal pain requiring the use of analgesics or fever with a body temperature of 37.5°C or higher that occurred after ESD without intraoperative or delayed perforation.^9^

### Colorectal ESD procedures

All patients underwent bowel preparation (polyethylene glycol, 2 L) before the colorectal ESD. The patients were sedated in the endoscopy room using intravenous injections of pethidine hydrochloride (35 mg/session) and midazolam (2–5 mg/session). Electrocardiographic monitoring was performed for each patient. We used a waterjet colonoscope (PCF‐Q260AZI/Q290ZI; Olympus Corporation) with a transparent hood (DH‐30CR for the scope tip; Fujifilm) attached to the tip of the scope. Marking, mucosal incision, and mucosal detachment were performed using a dual knife (KD‐655Q; Olympus Corporation) or a pro knife (ORISE ProKnife; Boston Scientific). We injected 0.4% sodium hyaluronic acid diluted in glycerol into the submucosal layer directly below the lesion and before mucosal detachment. A coagrasper (FD‐410LR; Olympus Corporation) and an endoscopic clip (HX‐610‐090; Olympus Corporation) were used to achieve hemostasis.

### Criteria for the discontinuation of medications for patients receiving antithrombotic medications

Patients taking at least one antithrombotic drug, regardless of the type, were considered to be taking antithrombotic drugs. In addition, the preoperative drug withdrawal period was defined in accordance with the *Gastrointestinal endoscopy treatment guidelines for patients taking antithrombotic drugs* (Japanese Gastroenterological Endoscopy Society 2017).^10^

### High‐frequency power supply (VIO3) settings

Table 1 lists the VIO3 settings used for colonic ESD at our facility. Mucosal incision was performed using the Endo Cut mode of VIO3 for both periods. Before changing the coagulation mode of VIO3 for submucosal layer dissection, Swift COAG (effect, 3.5 maximum; voltage, 1.1 kVp; power limitation, 105 W) was used. After the change, ESD was performed using the Swift COAG or Spray COAG mode (effect, 3.4 maximum; voltage, 4.3 kVp; power limit, 41 W). We first attempted to stop the intraoperative bleeding using an electrosurgical knife and coagulation.

**TABLE 1:** High‐frequency power supply (VIO3) settings.

	Cut setting				COAG setting	
	Mode	Effect	Duration	Interval	Mode	Effect
Mucosal incision	Endo Cut I	2	2	2	Swift COAG	3.5
Submucosal dissection	Endo Cut I	2	2	2	Swift COAG	3.5
Device hemostasis					Spray COAG	3.4
					Swift COAG	3.5
Hemostatic forceps					Soft COAG	5.5
Marking					Forced COAG	1

Before the settings were reviewed, the Swift COAG mode was used for submucosal dissection. However, the Spray COAG mode was used to perform hemostasis using an electrosurgical knife after the settings were reviewed. If it was difficult to stop the bleeding with an electrosurgical knife, the hemostatic forceps and Soft COAG modes were used.

### Primary endpoints

The primary endpoint was the change in the frequency of use of hemostatic forceps after the introduction of Spray COAG.

### Secondary endpoints

The secondary endpoints were the postoperative complications (postoperative bleeding, intraoperative perforation, delayed perforation, and PECS) after using Spray COAG.

### Statistical analysis

The heterogeneity of patient characteristics precluded a direct comparison of the frequencies of hemostatic forceps application before and after the introduction of Spray COAG without accounting for confounding variables. Consequently, the inverse probability of weighting (IPTW) method was used to mitigate baseline disparities between the cohorts.

The propensity scores (PSs) for each patient were estimated using a multivariable logistic regression model that incorporated variables such as age, medical practitioner proficiency, lesion site (colon/rectum), macroscopic type (LST‐G/NG), antithrombotic drug use, depth of invasion (Tis‐T1a/T1b), tumor length, and resection duration. The WeightIt package in R (version 4.2.3; The R Project for Statistical Computing) was used to implement the IPTW method to adjust for confounding factors across patient groups (available at https://cran.r‐project.org/web/packages/WeightIt/index.html).

A comparative analysis of patient characteristics before and after weighting by the inverse of the PS was conducted. The groups were considered homogeneous for each variable when the absolute standardized difference (ASD) was less than 0.1, in addition to other pertinent covariates. All statistical evaluations were performed using R software, with a two‐tailed significance threshold set at 5%.

## RESULTS

### Background factors before and after introducing Spray COAG

We compared age, sex, surgeon proficiency, tumor location, macroscopic type, antithrombotic drug administration rate, tissue type, invasion depth, tumor length, tumor invasion depth, pHM, pVM, pLy, pV, and resection time before and after the introduction of Spray COAG (Table 2). The age at the time of treatment was significantly older after the introduction of Spray COAG than before (66.7 ± 12.6 years versus 71.0 ± 11.7 years, *p* < 0.001). The major diameter of the tumor was significantly greater before the introduction of Spray COAG than after (30.7 ± 11.8 mm versus 28.1 ± 13.7 mm, *p* = 0.01). The ASD values were ≥0.1 for age, macroscopic type, tumor length, and resection time. After IPTW, all background factors had an ASD of <0.1, and no significant differences were observed for any background factor (Table 3). The AUC of the PS value calculated from this study dataset was 0.67.

**TABLE 2:** Patient background data and endoscopic and pathological factors (IPTW^†^, unadjusted).

	Before Spray COAG	After Spray COAG		
	*N* = 145	*N* = 162	*p*‐value	ASD
Age, mean ± SD	66.7 ± 12.6	71 ± 11.7	0.001	0.35
Sex (M/F)				
Male	74	88	0.57	0.05
Female	71	74		
Surgeon (%)				
Professional	95 (65%)	104 (64%)	0.81	0.02
Beginner	50 (35%)	58 (36%)		
Lesion site (%)				
Colon	108 (74%)	127 (78%)	0.50	0.06
Rectum	37 (26%)	35 (22%)		
Macroscopic, % (LST‐G/LST‐NG)				
LST‐G	100 (69%)	99 (61%)	0.19	0.11
LST‐NG	45 (31%)	63 (39%)		
Antithrombotic medication rate (%)				
Positive	28 (19%)	25 (15%)	0.46	0.05
Negative	117 (81%)	137 (85%)		
Secondary tissue type (%)				
adenoma	67(46%)	80 (49%)	0.66	0.04
cancer	78 (54%)	82 (51%)		
Depth of invasion (%)				
Tis‐T1a	73 (94%)	80 (98%)	0.36	0.03
T1b	5 (6%)	2 (2%)		
Tumor length (mm), mean ± SD	30.7 ± 11.8	28.1 ± 13.7	0.01	0.20
pHM (%)				
Positive	7 (5%)	5 (3%)	0.62	0.02
Negative	138 (95%)	157 (97%)		
pVM (%)				
Positive	1 (1%)	1 (1%)	0.83	0.01
Negative	144 (99%)	161 (99%)		
pLy (%)				
Positive	2 (3%)	1 (1%)	0.60	0.01
Negative	76 (97%)	81 (99%)		
pV (%)				
Positive	4 (5%)	0 (0%)	0.05	0.04
Negative	74 (95%)	82 (100%)		
Resection time (min), mean ± SD	62.5 ± 40.3	68.1 ± 42.8	0.14	0.14

^†^ Abbreviations: ASD, absolute standardized difference; F, female; IPTW, inverse probability of weighting; LST‐G, laterally spreading tumor granular type; LST‐NG, laterally spreading tumor non‐granular type; M, male; SD, standard deviation.

**TABLE 3:** Patient background data and endoscopic and pathological factors (IPTW^†^, adjusted).

	Before Spray COAG	After Spray COAG	*p*‐value	ASD
Age, mean ± SD	69.2 ± 12.4	68.4 ± 13.3	0.71	0.05
Sex (%)				
Male	157.8 (51.1)	169.4 (54.9)	0.53	0.01
Female	151.2 (48.9)	139.1 (45.1)		
Surgeon (%)				
professional	191 (61.8)	192.8 (62.5)	0.91	0.06
beginner	118.1 (38.2)	115.7 (37.5)		
Lesion site (%)				
colon	237.1 (76.7)	235.2 (76.2)	0.93	0.00
rectum	72.0 (23.3)	73.3 (23.8)		
Macroscopic (%)				
LST‐G	197.4 (63.9)	201.7 (65.4)	0.80	0.00
LST‐NG	111.7 (36.1)	106.8 (34.6)		
Antithrombotic medication rate (%)				
Positive	55.3 (17.9)	51.2 (16.6)	0.78	0.00
Negative	253.8 (82.1)	257.3 (83.4)		
Secondary tissue type (%)				
adenoma	138.0 (44.7)	157.0 (50.9)	0.30	0.00
cancer	171.0 (55.3)	151.5 (49.1)		
Depth of invasion (%)				
Tis‐T1a	301.9 (97.7)	301.0 (97.6)	0.96	0.01
T1b	7.2 (2.3)	7.5 (2.4)		
Tumor length (mm) ± SD	29.1 ± 11.4	29.6 ± 14.8	0.86	0.02
pHM (%)				
Positive	15.6 (5.0)	11.8 (3.8)	0.65	0.03
Negative	293.4 (95.0)	296.7 (96.2)		
pVM (%)				
Positive	1.6 (0.5)	3.1 (1.0)	0.62	0.01
Negative	307.5 (99.5)	305.4 (99.0)		
pLy (%)				
Positive	2.8 (0.9)	2.0 (0.6)	0.77	0.02
Negative	306.2 (99.1)	306.5 (99.4)		
pV (%)				
Positive	5.9 (1.9)	0 (0)	0.05	0.03
Negative	303.2 (98.1)	308.5 (100)		
Resection time, min ± SD	67.8 ± 45.2	66.4 ± 41.4	0.81	0.03

^†^ Abbreviations: ASD, absolute standardized difference; IPTW, inverse probability of weighting; LST‐G, laterally spreading tumor granular type; LST‐NG, laterally spreading tumor non‐granular type; SD, standard deviation.

### Comparison of the frequencies of use of hemostatic forceps before and after the introduction of Spray COAG

After IPTW, the frequency of use of hemostatic forceps decreased significantly after the introduction of Spray COAG (odds ratio [OR] = 0.12, 95% confidence interval [CI]: 0.06–0.23, *p* < 0.001; Table 4).

**TABLE 4:** Comparison of the odds ratio of hemostatic forceps use in the Spray COAG and non‐Spray COAG groups.^‡^

	IPTW adjusted	Odds ratio	95% CI**^‡^**	*p*‐value
Hemostatic forceps use	Unadjusted	0.10	0.05–0.18	<0.001
	Adjusted	0.12	0.06–0.23^‡^	<0.001

^‡^ Abbreviations: CI, confidence interval; IPTW, inverse probability of weighting.

### Comparison of the postoperative complications

The incidence of major complications, such as postoperative bleeding, intraoperative perforation, delayed perforation, and PECS, before and after the introduction of Spray COAG were compared. PECS significantly decreased after the introduction of Spray COAG (OR = 0.43, 95% CI: 0.22–0.88, *p* = 0.02). There were no significant differences in the incidence of postoperative bleeding, intraoperative perforation, or delayed perforation before and after the introduction of the Spray COAG (Table 5).

**TABLE 5:** Comparison of the risk of major complications in the Spray COAG and non‐Spray COAG groups.^†^

	IPTW adjusted	Odds ratio	95% CI**^‡^**	*p*‐value
**Major complications**				
Delayed bleeding	Unadjusted	0.42	0.16–1.05	0.07
	Adjusted	0.51	0.19–1.34^‡^	0.16
Perforation	Unadjusted	0.66	0.21–1.94	0.44
	Adjusted	0.37	0.12–1.21^‡^	0.15
Delayed perforation	Unadjusted	0.89	0.03–22.76	0.94
	Adjusted	0.83	0.05–13.68^‡^	0.90
PECS	Unadjusted	0.47	0.24–0.89	0.02
	Adjusted	0.43	0.22–0.88^‡^	0.02

^‡^ Abbreviations: CI, confidence interval; IPTW, inverse probability of weighting; PECS, post‐endoscopic submucosal dissection electrocoagulation syndrome.

## DISCUSSION

The introduction of Spray COAG dramatically improved the hemostatic performance of electrocautery in colorectal ESD and reduced the frequency of use of hemostatic forceps. To our knowledge, this is the first study to examine the effectiveness of Spray COAG in VIO3 for colonic ESD. High‐frequency surgical equipment, such as VIO3, is indispensable for colorectal ESD. The output of the high‐frequency surgical device has incision and coagulation modes. For the incision mode, the Joule heat generated by a high‐frequency current (continuous wave) was continuously outputted, which rapidly increased the temperature of the intracellular fluid and generated a steam explosion. In contrast, a high‐frequency current (intermittent waves) was applied intermittently to slowly increase the temperature of intracellular and extracellular fluids, thereby dehydrating and drying the tissue.^11^

Morita et al. recommended Swift COAG or Forced COAG as coagulation modes for colon ESD using a VIO300D high‐frequency surgical device.^11^ For VIO3, Maehara et al. are currently conducting a multicenter randomized controlled trial comparing the hemostatic ability of spray and forced COAG for ESD of upper gastrointestinal tumors.^12^

In this study, the frequency of hemostatic forceps use decreased significantly after introducing Spray COAG. On the basis of this, we believe that Spray COAG improves the hemostatic ability of the electrosurgical knife. The maximum voltage peak (Vp) value for Swift COAG was set to 2500 Vp, and the submucosal layer, which had a low resistance value, was dissected with high power. The system suppresses output and performs coagulation in areas with high resistance, such as the blood vessels and blood. Therefore, the output decreases when the tissue resistance reaches 200–300 Ω (Figure S1A).^13^ However, a sufficient hemostatic effect was not obtained because of excessive restriction of the output due to the rapid change in the resistance value when active bleeding was detected. The maximum Vp value for Spray COAG was 4300 Vp, which was higher than that for Swift COAG. The output did not decrease regardless of the tissue resistance value, and it was considered that a hemostatic effect was obtained, even for highly active bleeding (Figure S1B).^13^

PECS is characterized by temporary inflammation that occurs when electrocoagulation heat is transmitted deep into the muscle layer at the resection site during endoscopic treatment. Ochi et al. demonstrated a correlation between the total amount of Joule heat on a dissected surface and the onset of PECS.^14^ Spray COAG has a high output setting, and there were concerns about the increase in the incidence of PECS with an increase in the amount of Joule heat applied to the dissected surface; however, after introducing Spray COAG, the PECS incidence decreased significantly. Spray COAG discharges electricity without contacting the dissected surface, and it is assumed that heat conduction to the deep muscle layer was reduced while ensuring hemostasis on the surface of the ulcer.

In this study, no significant increase in the incidence of major ESD‐related complications, such as intraoperative or delayed perforation, was observed after the introduction of Spray COAG. We believe that the safety of the electrosurgical knife and Spray COAG is equivalent to or higher than that of Swift COAG.

The possible benefits of introducing Spray COAG are as follows. First, it reduces the frequency of use of hemostatic forceps, making it possible to reduce ESD costs. Medical equipment for advanced treatments such as ESD is expensive. We believe that cost reduction is important not only from a medical and economic perspective but also for the widespread adoption of ESD globally. Second, by reducing the frequency of endoscopic device replacements, bleeding can be stopped without losing sight of the bleeding point. In the conventional method, hemostatic forceps are used when it is difficult to stop the bleeding with an electrosurgical knife. Therefore, the electrosurgical knife is removed, and hemostatic forceps are inserted to stop the bleeding. During this time, bleeding from the ruptured blood vessel continues, and the bleeding point is often lost after hemostatic forceps are inserted. For Spray COAG, it is only necessary to change the VIO3 settings when bleeding is confirmed. Therefore, it is possible to stop the bleeding without losing sight of the bleeding point, which we believe will lead to less fatigue for the surgeon.

It was anticipated that a decrease in the frequency of hemostatic forceps usage would lead to a reduction in procedure time. However, in this study, despite the decrease in the frequency of hemostatic forceps usage after the introduction of Spray COAG, no significant difference in resection time was observed. Although the frequency of hemostatic forceps usage was not measured in this study, it is suggested that the incidence of encountering severe pulsatile bleeding is relatively low in colonic ESD compared to gastric ESD. The possibility is suggested that when the frequency of hemostatic forceps usage is low, the time required to switch between the hemostatic forceps and the electrosurgical knife is relatively short, resulting in no significant difference in treatment time.

## LIMITATIONS

As this was a single‐center retrospective study, the sample was small. The findings of this study may not be generalizable to other settings because of the differences in patient populations, surgeon skill levels, and institutional protocols. Although propensity methods helped adjust for several confounders, there may still be unmeasured confounding variables that could influence the results. The length of follow‐up for complications was not the same for all patients, and delayed‐ and long‐term complications were not evaluated. Future multicenter prospective studies may resolve these issues. Although not evaluated in this study, we believe that the use of Spray COAG reduces the frequency of hemostatic forceps use, thus reducing the cost of hemostatic forceps. Therefore, a cost‐effectiveness analysis comparing Spray COAG with conventional hemostatic methods is needed.

In this study, the frequency of hemostat use was not measured. In the future, measuring the frequency of hemostat use and the duration of electrocautery may clarify the correlation between the use of hemostats and PECS.

Moreover, the PS calculated from the dataset of this study was 0.67, which was less than 0.8. This is a limitation of the study, and further research, such as prospective trials, is needed.

## CONCLUSION

Spray COAG reduced the frequency of hemostatic forceps use in colorectal ESD. Spray COAG may have contributed to reducing the frequency of hemostatic forceps use and the incidence of PECS during colorectal ESD.

## CONFLICT OF INTEREST STATEMENT

Hisatomo Ikehara has received lecture fees from Mundiphama Co., AI Medical Service Inc., Olympus Co., and FUJIFILM Co. Chika Kusano has received lecture fees from Takeda Pharmaceutical Co., EA Pharma Co., 3‐D Matrix Ltd., Boston Scientific Co., Olympus Marketing Inc., FUJIFILM Medical Co., and FUJIFILM Co.

## ETHICS STATEMENT

This study was approved by the Ethics Review Board of the Kitasato University School of Medicine and Hospital (approval number: B22‐228).

## PATIENT CONSENT STATEMENT

The opt‐out method was used to obtain consent from the study participants.

## Supporting information


**FIGURE** S**1** (a) Output diagram of Swift COAG at VIO3. (b) Output diagram of Spray COAG at VIO3.

## Data Availability

All data generated or analyzed during this study are included in this published article.
