# Supplementary figures and images for: Effect of spray COAG mode on hemostasis in colorectal endoscopic submucosal dissection using inverse probability of treatment weight analysis
Source: DEN Open. 2024 Sep 16;5(1):e70008. doi: 10.1002/deo2.70008 (PMC11405631; doi:10.1002/deo2.70008)

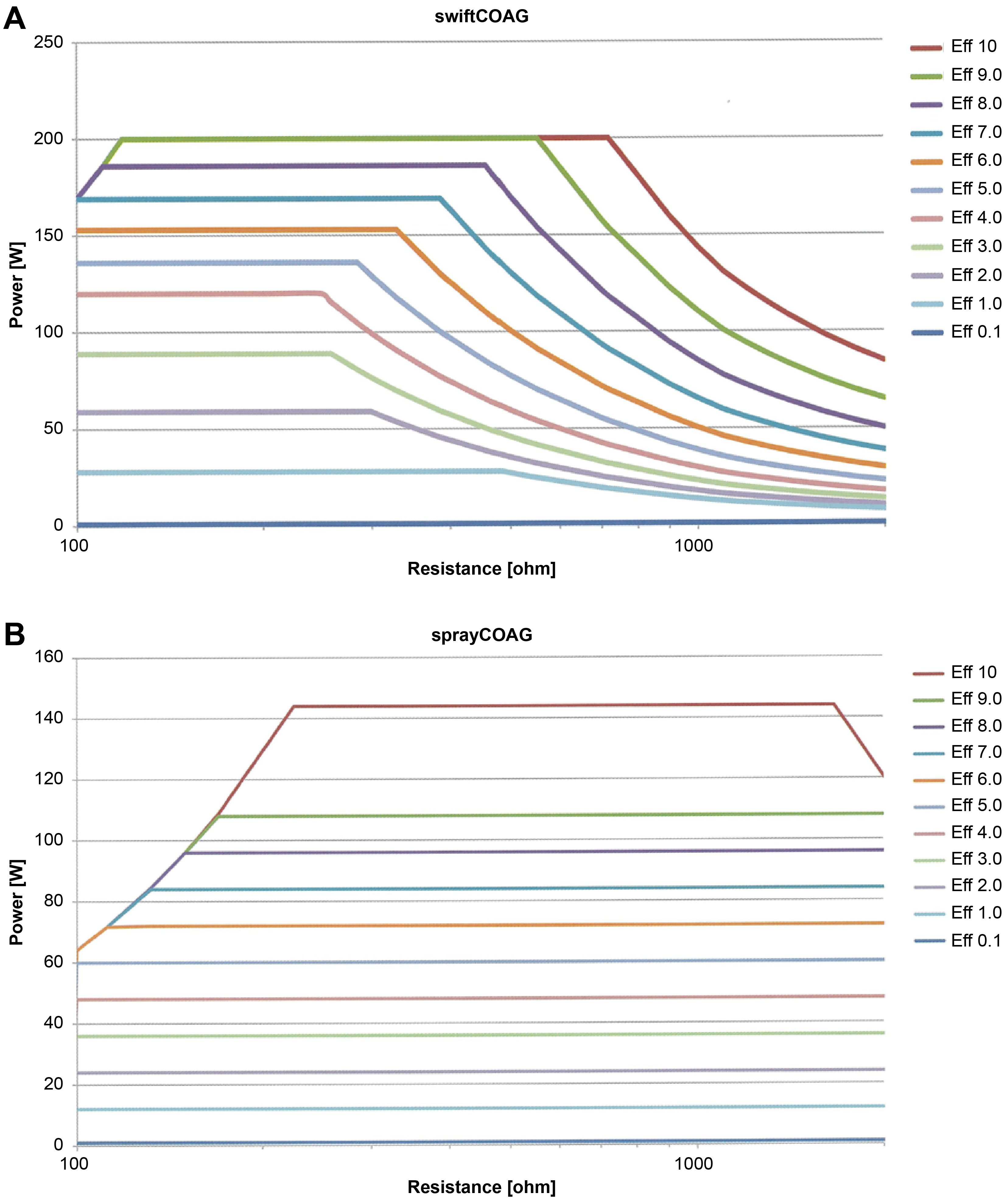

Supplement: Supplementary file 1 — FIGURE S1 (a) Output diagram of Swift COAG at VIO3. (b) Output diagram of Spray COAG at VIO3. [file DEO2-5-e70008-s001.tif]
